# Nitrite of Amyl in Nervous Cephalalgia

**Published:** 1876-01

**Authors:** 


Nitrite of Amyl in Nervous Cephalalgia.—In the December number of the *London Lancet*, R. A. Douglas-Lithgow, L.R.C.P., etc., gives his experience with the nitrite amyl in nervous headache. He gives two drops by inhalation. He contends that when due care is taken in the selection of cases, he has never known it to fail to produce entire and almost immediate relief. He says: I place two drops on the palm of the patient's hand, and quickly diffusing these with my finger over the palmer surface, I tell her to cover her mouth and nose with her hand, and to inspire deeply and quietly. No time should be lost after the nitrite is dropped on the hand, as it evaporates rapidly. The patient should be seated while inhaling, as the peculiar effects of the nitrite are produced almost instantaneously, and may occasionally alarm a very nervous or hysterical female. Fortunately these symptoms last a very short time—generally less than two or three minutes—and with their cessation the pain almost invariably ceases. Two drops may be given as a draught in water, instead of by inhalation, but I have found the latter mode much more satisfactory. * * *

Used as I have just recommended, I don't think there is the slightest risk in its administration; but, owing to the temporary palpitation of the heart produced in most patients, care should be taken in administering it in cases of organic cardiac disease, etc.

